# Trends in Screening for Social Risk in US Physician Practices

**DOI:** 10.1001/jamanetworkopen.2024.53117

**Published:** 2025-01-03

**Authors:** Amanda L. Brewster, Hector P. Rodriguez, Genevra F. Murray, Valerie A. Lewis, Karen E. Schifferdecker, Elliott S. Fisher

**Affiliations:** 1 Division of Health Policy and Management, School of Public Health, University of California, Berkeley, Berkeley; 2 Department of Public Health Policy and Management, School of Global Public Health, New York University, New York; 3 Department of Health Policy and Management, UNC Gillings School of Global Public Health, University of North Carolina at Chapel Hill, Chapel Hill; 4 The Dartmouth Institute for Health Policy and Clinical Practice, Geisel School of Medicine at Dartmouth, Lebanon, New Hampshire

## Abstract

**Importance:**

A wealth of research on screening for social risks in health care has emerged, but evidence is lacking on how social risk screening among physician practices has changed over time.

**Objectives:**

To evaluate trends in screening for social risks among US physician practices and examine practice characteristics associated with adoption of social risk screening.

**Design, Setting, and Participants:**

The main analysis used a repeated cross-sectional design to analyze results from US physician practices that completed the National Survey of Healthcare Organizations and Systems, a nationally representative survey of physician practices, in 2017 and 2022. Sensitivity analysis tested robustness in a cohort of practices that completed surveys in both years.

**Exposures:**

Survey year and characteristics of physician practices.

**Main Outcomes and Measures:**

Whether physician practices systematically screened patients for 5 common social risks: food, housing, utilities, interpersonal violence, and transportation.

**Results:**

In this cross-sectional study, 3442 practice survey responses were studied. Weighted results showed that in 2022, 27% (95% CI, 23%-32%) of practices reported screening for all 5 of the social risks, an increase from 15% (95% CI, 13%-18%) of practices in 2017 (*P* < .001). Unadjusted results showed a significant increase in the mean number of social risks screened per practice, from 1.71 (95% CI, 1.60-1.82) in 2017 to 2.34 (95% CI, 2.12-2.55) in 2022. Practice characteristics associated with screening for more social risks remained consistent between 2017 and 2022; these included being a federally qualified health center (incidence rate ratio [IRR], 1.550 [95% CI, 1.336-1.799]) and having higher innovation culture scores (IRR, 1.012 [95% CI, 1.010-1.015]), higher advanced information system scores (IRR, 1.003 [95% CI, 1.001-1.005]), and higher payment reform exposure scores (IRR, 1.002 [95% CI, 1.000-1.003]).

**Conclusions and Relevance:**

In this survey-based cross-sectional study of US physician practices, social risk screening increased substantially from 2017 to 2022, although still less than one-third of practices systematically screened for a set of 5 common social risks. What remains to be seen is whether practices use these data to help improve patient health by adjusting health care or referring patients for assistance with social needs. As policies and incentives increasingly emphasize social risk screening, it will be important to assess the association of screening and referrals with patient outcomes.

## Key Points

**Question:**

Has social risk screening in US physician practices changed over time?

**Findings:**

This cross-sectional study found that in 2022, 27% of physician practices reported systematically screening patients for 5 common social risks, a significant increase from 15% in 2017.

**Meaning:**

This study suggests that social risk screening by physician practices increased substantially from 2017 to 2022, with similar increases across different types of practices.

## Teaser

This cross-sectional study evaluates trends in screening for social risks among US physician practices from 2017 to 2022 and examines practice characteristics associated with adoption of social risk screening.

## Introduction

A wealth of research on screening for social risks in health care has emerged in recent years,^1,2^ accelerating after the National Academies of Science, Engineering, and Medicine put screening at the center of its recommendations for improving the integration of social care with health care.^3,4^ However, to our knowledge, the last nationally representative data on the extent to which physician practices systematically screen their patients for social risks came from 2017, in the National Survey of Healthcare Organizations and Systems (NSHOS). The 2017 NSHOS showed that just over half of physician practices that provide primary care were systematically screening patients for interpersonal violence (56.4%) and less than half of practices were screening for 4 other social risks (35.4% for transportation needs, 29.6% for food insecurity, 27.8% for housing instability, and 23.1% for utility needs).^5^

It is unknown how screening processes have changed among physician practices since 2017, although data on social risk screening by hospitals show that by 2022, 53% to 72% of hospitals were screening for each of these 5 social risks, and mostly commonly using the data for discharge planning.^6^ To evaluate how social risk screening has changed among physician practices in recent years, we used data from the second NSHOS, fielded in 2022, to assess trends in systematically screening patients for social needs between 2017 and 2022 and practice characteristics associated with uptake of social risk screening.

As the evidence base on implementation of social risk screening in health care has increased in recent years—documented in systematic reviews^1,2,7^—more physician practices may have adopted social risk screening. The types of practices implementing social risk screening could also be changing over time, particularly as national standards-setting bodies introduce^8^ or consider^9^ recommendations on the subject. Based on prior research,^10^ we hypothesized that the prevalence of social risk screening would have increased more substantially between 2017 and 2022 among practices caring for a high share of Medicaid beneficiaries because such practices may expect to identify relatively high levels of social risk in their patient populations. With social risk screening remaining a relatively novel component of health care, we also hypothesized that practices with an organizational culture conducive to innovation would remain more likely to screen for social risks, as was the case in 2017.

## Methods

### Sample and Data Collection

Using a repeated cross-sectional design, we analyzed both waves of the NSHOS data—from 2017 and 2022—on screening patients for social risks and on practice characteristics. The NSHOS is a nationally representative survey of physician practice sites that include 3 or more adult primary care physicians.^11^ In both NSHOS waves, the survey was completed by a practice leader on behalf of the practice. The OneKey database, produced by IQVIA, was used to identify physician practices and draw a representative national sample. In 2017, a total of 2333 of 4976 physician practices completed the NSHOS survey (response rate, 46.9%), and after removing responses from practices that were identified as ineligible or missing variables needed for our analysis, survey responses from 2190 practices were available for analysis. In 2022, survey responses were collected from 1252 of 3498 physician practices (response rate, 35.8%). Some survey items changed from 2017 to 2022; to allow comparison over time, we used only the items that were asked in both survey waves. Study procedures were reviewed and approved by the Committee for Protection of Human Subjects of the University of California, Berkeley. Individuals completing the NSHOS on behalf of their practice provided written consent to complete the survey. This cross-sectional study followed the Strengthening the Reporting of Observational Studies in Epidemiology (STROBE) reporting guideline.

### Dependent Variable

The NSHOS survey asked practices to report whether they had systems in place to screen for 5 social risks: food insecurity, housing instability, utility needs, interpersonal violence, and transportation needs. The survey question began “Does your practice have a system in place to routinely screen patients for:” and respondents selected “yes” or “no” for each potential risk. As a measure of screening intensity, we calculated the number of social risks screened by each practice (range, 0-5) and whether the practice screened for all 5 risks.

### Independent Variables

#### Year

Practice survey responses were identified as being from either 2017 or 2022.

#### Share of Practice Revenue From Medicaid

Practice respondents estimated the share of their practice’s revenue from Medicaid. This variable could fall into 5 categories, ranging from less than 10% to more than 80%.

#### Culture of Innovation

The NSHOS contained a 5-item scale representing perceptions of the practice’s culture of innovation, such as whether it is common to try new ideas and approaches, publicize care innovations, share challenges, and have protected time for innovation (range, 0-100). Details of scales are provided in the eAppendix in Supplement 1.

#### Advanced Information Systems

The NSHOS contained a 6-item scale representing the capacity of the practice’s information systems (eg, whether it has connection with hospital information systems, patient access to records, messaging capabilities, prescription fill reports, and advanced analytic systems) (range, 0-100).

#### Exposure to Value-Based Payment Models

The NSHOS contained a 4-item scale representing the practice’s exposure to value-based payment models through participation in capitated contracts and different types of accountable care organizations (Medicare, Medicaid, and commercial) (range, 0-100).

### Covariates

Additional NSHOS survey items measured other practice characteristics that could be associated with diffusion of care delivery innovations, including practice ownership, size, and geographic region, which were included as covariates in the regression models for repeated cross-sectional analyses.

### Statistical Analysis

We calculated response-weighted descriptive statistics for the 2017 and 2022 survey respondents and used χ^2^ tests and *t* tests to examine differences between the 2 years. The main analysis used multivariable Poisson regression modeling to evaluate the association of survey year and practice characteristics with the count of social risks screened by physician practices (possible range, 0-5 risks). For our primary specification, we used a repeated cross-sectional design, including all physician practices that responded to the NSHOS in 2017, 2022, or both years, to include the maximum sample size in primary analyses (3442 survey responses from 2728 distinct practices, of which 714 practices had responses in both years). All analyses used survey sampling and nonresponse weights, and SEs were clustered by practice identifier to account for practices with survey responses in both years. Model results were used to calculate the estimated marginal mean values for the selected practice characteristics, using the Stata Margins command in Stata, version 15 (StataCorp LLC). Throughout results and reporting, 95% CIs were used. All *P* values were from 2-sided tests, and results were deemed statistically significant at *P* < .05.

We conducted several sensitivity analyses to test the robustness of our findings. First, we limited the sample to the cohort of 714 practices with survey responses in both years and repeated the analysis using fixed effects for practice (ie, comparing each practice with itself at 2 time points to examine how change in social risk screening was associated with change in other time-variant practice characteristics from 2017 to 2022). Second, we tested interactions between year and practice characteristics in our main repeated cross-section models to understand how changes in the characteristics of practices in our sample from 2017 to 2022 may have influenced results. Third, we used logistic regression models to examine screening for each of the 5 social risks separately to examine potential heterogeneity by social risk factor.

## Results

Our sample included a total of 3442 practice survey responses (2190 from 2017 and 1252 from 2022). As shown in Table 1, most surveys in the sample (2773 of 3442 [81%]) came from practices that had 12 or fewer physicians. Weighted results showed that the most common ownership model for practices in the sample was being owned by a larger health care system (2017, 30% [95% CI, 27%-32%]; 2022, 39% [95% CI, 33%-45%]), followed by being independently owned (2017, 29% [95% CI, 26%-32%]; 2022, 20% [95% CI, 17%-23%]). A total of 966 of 3442 survey responses (28%) came from practices that received 20% or more of their practice revenue from Medicaid. Weighted characteristics of physician practices changed slightly between the 2017 and 2022 samples, with significantly different distributions in practice revenue from Medicaid, practice ownership, practice size, and US Census region of practices. On average, practices experienced significant decreases in innovation culture from 2017 to 2022 and significant increases in advanced information system capabilities and payment reform participation.

**Table 1.** Characteristics of Sampled Practices (Response Weighted)

Characteristic	2017		2022		*P* value^a^
	Raw total No. (n = 2190)	Weighted % (95% CI)	Raw total No. (n = 1252)	Weighted % (95% CI)	
Social risk screening category					
Food insecurity	613	29 (27-32)	592	47 (42-52)	<.001
Housing instability	587	28 (25-30)	557	44 (39-49)	<.001
Utility needs	483	23 (20-26)	427	34 (29-39)	<.001
Interpersonal violence	1247	56 (53-59)	740	61 (56-66)	.11
Transportation needs	752	35 (32-38)	600	47 (42-53)	<.001
All 5 social risks	346	15 (13-18)	335	27 (23-32)	<.001
Any of the 5 social risks	1445	67 (64-69)	911	74 (69-79)	.007
Share of practice revenue from Medicaid, %					
<10	1202	55 (52-58)	602	45 (40-51)	<.001
10-19	402	16 (14-19)	270	25 (20-30)	
20-49	451	22 (20-25)	238	22 (17-27)	
50-79	117	6 (5-8)	102	6 (5-8)	
≥80	18	1 (0-1)	40	3 (2-4)	
Practice ownership					
Independently owned	565	29 (26-32)	364	20 (17-23)	<.001
Larger physician group	243	9 (8-11)	111	6 (5-9)	
Hospital	285	13 (11-15)	106	14 (11-19)	
Health care system	733	30 (27-32)	383	39 (33-45)	
FQHC	315	16 (14-19)	264	20 (16-23)	
Other or missing	49	3 (2-4)	24	1 (1-2)	
Practice size, No. of physicians					
≤3	696	37 (34-40)	415	33 (28-38)	.004
4-7	773	33 (30-36)	468	40 (35-45)	
8-12	259	10 (9-12)	162	14 (10-18)	
13-19	108	5 (4-7)	59	3 (2-4)	
≥20	354	14 (12-16)	148	11 (8-14)	
US region					
Northeast	433	21 (19-24)	241	21 (17-26)	.02
Midwest	631	25 (23-27)	336	32 (27-37)	
South	578	29 (26-32)	368	28 (24-33)	
West	548	25 (22-28)	307	19 (15-22)	

Abbreviation: FQHC, federally qualified health center.

^a^ *P* values for statistical significance at 95% confidence of differences between 2017 and 2022, based on χ^2^ tests for proportions.

**Figure 1.**
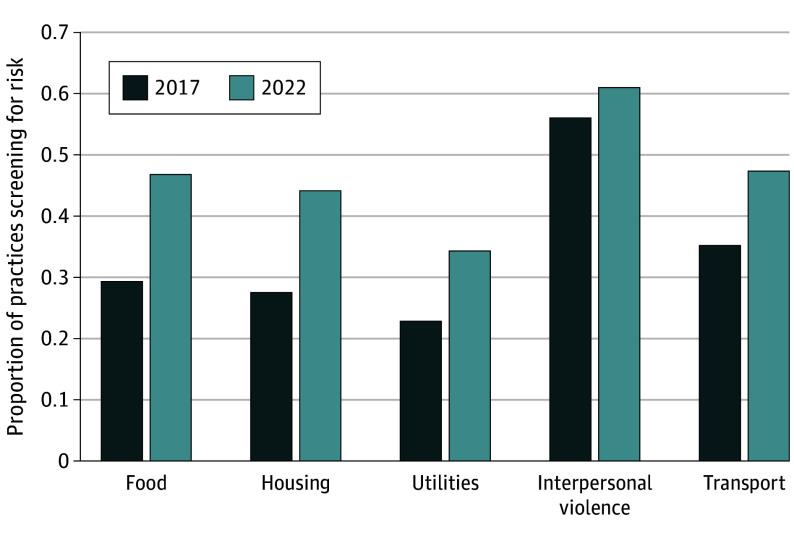
Proportion of Practices Screening for Specified Social Risks (Weighted), 2017-2022 The proportion of practices screening for food insecurity, housing instability, utility needs, and transportation needs increased significantly from 2017 to 2022 (3442 practice survey responses).

Unadjusted results indicated a substantial increase in screening for social risks by physician practices from 2017 to 2022 (Figure 1), with the proportion screening for food insecurity increasing from 29% (95% CI, 27%-32%) to 47% (95% CI, 42%-52%) (*P* < .001), screening for housing instability increasing from 28% (95% CI, 25%-30%) to 44% (95% CI, 39%-49%) (*P* < .001), screening for utility needs increasing from 23% (95% CI, 20%-26%) to 34% (95% CI, 29%-39%) (*P* < .001), and screening for transportation needs increasing from 35% (95% CI, 32%-38%) to 47% (42%-53%) (*P* < .001). The proportion screening for interpersonal violence increased from 56% (95% CI, 53%-59%) to 61% (95% CI, 56%-66%), although this increase was not statistically significant (*P* = .11). The share of practices screening for all 5 social risks increased from 15% (95% CI, 13%-18%) in 2017 to 27% (95% CI, 23%-32%) in 2022 (*P* < .001), while the share of practices screening for any of these 5 risks increased from 67% (95% CI, 64%-69%) to 74% (95% CI, 69%-79%; *P* = .007). As reported in Table 2, the mean number of social risks screened increased from 1.71 (95% CI, 1.60-1.82) risk factors in 2017 to 2.34 (95% CI, 2.12-2.55) risk factors in 2022. Among practices screening for any social risks, the mean number of risk factors screened increased from 2.57 (95% CI, 2.45-2.69) in 2017 to 3.14 (95% CI, 2.92-3.37) in 2022 (*P* = .007).

**Table 2.** Characteristics of Sampled Practices (Response Weighted), Continuous Variables

Characteristic	2017 Weighted mean (95% CI)	2022 Weighted mean (95% CI)	*P* value^a^
Count of social risk screenings (of 5)	1.71 (1.60-1.82)	2.34 (2.12-2.55)	<.001
Innovation culture scale score	52.60 (51.35-53.84)	42.85 (40.29-45.40)	<.001
Advanced information systems score	51.55 (50.09-53.00)	61.32 (59.21-63.43)	<.001
Payment reform exposure score	39.03 (37.12-40.94)	55.56 (51.53-59.60)	<.001

^a^ *P* values for statistical significance at 95% confidence of differences between 2017 and 2022, based on *t* tests.

**Figure 2.**
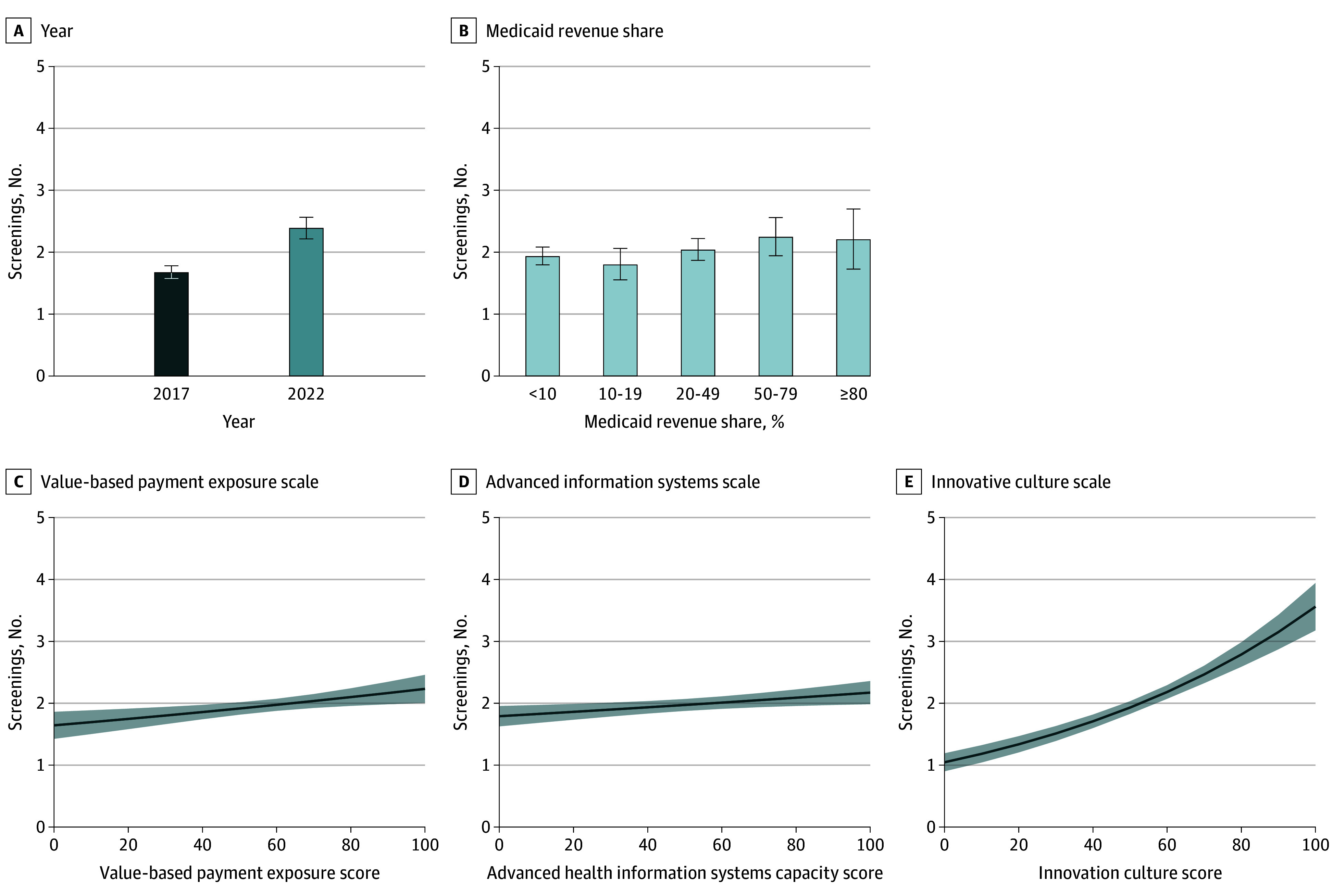
Adjusted Estimated Counts of Social Risks Screened (Margins) by Year and Practice Characteristics Graphs reflect estimated counts of social risks screened at varying levels of selected independent variables, based on the fully adjusted Poisson model presented in Table 3. In panels A and B, 95% CIs are shown with error bars, and in panels C, D, and E, 95% CIs are shown with shading (3442 practice survey responses).

As reported in Table 3, fully adjusted estimates indicate that the count of social risks screened per practice increased from 2017 to 2022 (IRR, 1.423 [95% CI, 1.297-1.561]). Practice characteristics associated with increased social risk screening included being a federally qualified health center (FQHC) (IRR, 1.550 [95% CI, 1.336-1.799]; *P* < .001) and having higher innovation culture scores (IRR, 1.012 [95% CI, 1.010-1.015]; *P* < .001), higher advanced information system scores (IRR, 1.003 [95% CI, 1.001-1.005]; *P* = .005), and higher payment reform exposure scores (IRR, 1.002 [95% CI, 1.000-1.003]; *P* = .01). Practices located in the South Census region had less social risk screening compared with the reference category (Northeast) (IRR, 0.867 [95% CI, 0.755-0.996]; *P* = .04). To facilitate interpretation of the fully adjusted results, Figure 2 graphs estimated marginal mean values of the count of social risk screenings associated with year and across a range of values for specified practice characteristics (marginal mean values are provided in eTable 1 in Supplement 1). The estimated marginal mean count of social risks screened in 2017 was 1.68 (95% CI, 1.59-1.78), which increased to 2.39 in 2022 (95% CI, 2.21-2.58). The scores for innovation culture, advanced information systems, and payment reform exposure were scaled to run from 0 to 100, where 0 indicates the lowest possible score and 100 indicates the highest possible score. Figure 2C, D, and E illustrate the count of social risk screenings estimated across the range of potential scale scores.

**Table 3.** Practice Characteristics Associated With Count of Social Risks Screened in 2017 and 2022 (3442 Practice Survey Responses)

Characteristic	IRR (95% CI)^a^	*P* value
Year		
2017	1 [Reference]	NA
2022	1.423 (1.297-1.561)^b^	<.001
Share of practice revenue from Medicaid, %		
<10	1 [Reference]	NA
10-19	0.932 (0.797-1.090)	.38
20-49	1.054 (0.939-1.183)	.37
50-79	1.161 (0.989-1.364)	.07
≥80	1.141 (0.900-1.447)	.28
Practice owned by		
Independently owned	1 [Reference]	NA
Larger physician group	1.043 (0.874-1.246)	.64
Hospital	1.162 (0.976-1.384)	.09
Health care system	1.047 (0.911-1.203)	.52
FQHC	1.550 (1.336-1.799)^b^	<.001
Other or missing	1.158 (0.853-1.573)	.35
Practice size, No. of physicians		
≤3	1 [Reference]	NA
4-7	0.934 (0.831-1.050)	.26
8-12	1.019 (0.867-1.198)	.82
13-19	0.957 (0.804-1.140)	.63
≥20	1.036 (0.896-1.197)	.63
Region		
Northeast	1 [Reference]	NA
Midwest	0.996 (0.861-1.152)	.96
South	0.867 (0.755-0.996)^c^	.04
West	0.934 (0.815-1.070)	.32
Innovation culture score	1.012 (1.010-1.015)^b^	<.001
Advanced information systems score	1.003 (1.001-1.005)^d^	.005
Payment reform exposure score	1.002 (1.000-1.003)^c^	.01

Abbreviations: FQHC, federally qualified health center; IRR, incidence rate ratio; NA, not applicable.

^a^ IRR values are exponentiated coefficients that reflect the change in count of social risk screenings per practice associated with change in the independent variables. Reported screenings can range from 0 to 5. A total of 2728 unique practices ever responded to the survey, 714 of which responded in both 2017 and 2022. Standard errors are clustered by practice in the models to account for practices that responded in both years.

Sensitivity analyses using fixed-effects models to examine change over time in the subset of practices with survey responses in both years showed largely similar associations between time-varying characteristics and social risk screening, although exposure to payment reform was not significant (eTable 2 in Supplement 1). Sensitivity analyses examining interactions of year and independent variables—as an alternative approach to testing whether practice characteristics associated with social risk screening changed over time—found none of the interaction terms to be significant (eTable 3 in Supplement 1). Sensitivity analyses that examined the probability of screening for each of the 5 social risks also showed generally similar characteristics associated with a higher likelihood of screening as the main model, except advanced information systems and exposure to payment reform were not significantly associated with screening for utilities or transportation, and exposure to payment reform was not significantly associated with screening for interpersonal violence (eTable 4 in Supplement 1).

## Discussion

Social risk screening by physician practices increased significantly from 2017 to 2022, although still less than one-third of practices systematically screen for a set of 5 common social risks. This marked increase suggests that practices nationwide are changing processes of care to more systematically consider patients’ social circumstances, as has been recommended by many leading health care institutions over the past 5 years and advanced as a pillar of the Biden Administration’s Playbook to Address Social Determinants of Health.^4,12,13^ Screening for social risks does not necessarily imply that practices are using the information to either adjust care or provide referrals to address social needs, but it is normally the first step toward doing so. The increase in social risk screening suggests that US physician practices have become better positioned to recognize and potentially address unmet social needs in vulnerable patient populations.

Certain types of practices screened for more social risks in both time periods, including FQHCs, practices with more innovative cultures, practices with advanced information systems, and practices exposed to more payment reforms. However, in contrast to our expectation that an increase in social risk screening from 2017 to 2022 would be concentrated in practices serving patients with lower income—such as FQHCs and practices caring for a relatively high share of Medicaid patients—the increases in social risk screening was not limited to these practices. These patterns suggest that social risk screening is becoming more common in primary care practice settings of various types.

Despite a substantial decrease in innovation culture scores over time, having a practice culture conducive to innovation was nonetheless strongly associated with greater adoption of social risk screening. Detrimental associations of the COVID-19 pandemic with health care worker burnout and retention have been well documented,^14,15,16^ and the associations of the COVID-19 pandemic with staffing shortages, time strain, and motivation may explain the decrease in innovation culture. Absent the pandemic, it is possible that more practices have been able to experiment with social risk screening. With best practices for implementing social risk screening still being established, physician practices need to work through questions about which screening tools to use as well as what modalities and staffing models to deploy, areas where limited evidence is available to guide decision-making.^1^ Fostering innovative primary care practice cultures, which provide time and resources to test and disseminate innovative care processes, could further encourage social risk screening. Growth and sustainment of social risk screening will need to take account of competing demands on already stretched primary care clinicians.^17^

### Limitations

This study has some limitations. First, our main specification used a repeated cross-sectional design, so our findings on changes over time could have been influenced by differing practices in the 2017 vs 2022 samples. We used sampling and nonresponse response weights to address this potential bias. Furthermore, our results remained consistent when fixed practice effects were used to examine the practices that responded in both 2017 and 2022, supporting the main results. Second, our survey-based measure of social risk screening does not allow for an in-depth insight into how practices are implementing social risk screening, and longitudinal practice data are not available to assess practice referral and care delivery processes associated with addressing unmet social needs. Social risk screening, however, is a foundational practice capability needed to improve referral and service delivery. Third, although NSHOS surveys were typically completed by a single practice leader, the respondent may not have known about every aspect of the practice assessed, leading to potential misclassification. Measurement error would likely bias our results toward the null, suggesting that our study results may represent lower bounds. Fourth, NSHOS survey response rates, at 38.4% in 2022 and 46.9% in 2017, could limit the generalizability of findings to all US physician practices in the event that respondents were not representative, although sampling and nonresponse weights help address this issue.

## Conclusions

Our results provide evidence that social risk screening increased substantially in recent years. What remains to be seen is whether practices can use the new information they are gathering about patients’ social risks to help improve their health and health care. It will be important to continue tracking screening as well as the work by practices to assist patients with identified social needs or adjust health care based on social needs. As policies and programs that support social care integration into health care continue to be tested and disseminated, it will be important to examine how social risk screening, referral, and service delivery processes are associated with patient outcomes.

## Notes

Supplement 1.
eAppendix.
**eTable 1.** Marginal Mean Count of Social Risks Screened From Main Model **eTable 2.** Sensitivity Analysis 1 **eTable 3.** Sensitivity Analysis 2 **eTable 4.** Sensitivity Analysis 3

Supplement 2.
Data Sharing Statement
