# Supplementary material for: Trends in Screening for Social Risk in US Physician Practices
Source: JAMA Netw Open. 2025 Jan 3;8(1):e2453117. doi: 10.1001/jamanetworkopen.2024.53117 (PMC11699528; doi:10.1001/jamanetworkopen.2024.53117)
Supplement: Supplement 1. — eAppendix. eTable 1. Marginal Mean Count of Social Risks Screened From Main Model eTable 2. Sensitivity Analysis 1 eTable 3. Sensitivity Analysis 2 eTable 4. Sensitivity Analysis 3 [file jamanetwopen-e2453117-s001.pdf]

# Supplementary Online Content

Brewster AL, Rodriguez HP, Murray GF, Lewis VA, Schifferdecker KE, Fisher ES. Trends in screening for social risk in US physician practices. *JAMA Netw Open.* 2025;8(1):e2453117. doi:10.1001/jamanetworkopen.2024.53117

**eAppendix.**
**eTable 1.** Marginal Mean Count of Social Risks Screened From Main Model
**eTable 2.** Sensitivity Analysis 1
**eTable 3.** Sensitivity Analysis 2
**eTable 4.** Sensitivity Analysis 3

This supplementary material has been provided by the authors to give readers additional information about their work.

**eAppendix.**

**Construction of Composite Scales**

NSHOS survey responses were used to generate 3 composite scales measuring: (1) Culture of innovation (5-item scale), (2) Advanced information system capacity (7-item scale), and (3) Exposure to value-based payment. The survey items and scoring methods used calculate scale scores were as follows:

**(1) Culture of innovation**. Respondents used a Likert scale to indicate how often the following things happen in the practice. (Answer choices: Never, Sometimes, Often, Always).

- Successful care delivery innovations are highly publicized within the practice
- Team members openly share patient care challenges and failures with each other
- There is protected time given to generate new ideas and innovations
- We encourage trying new ideas to see if they work
- We consider ourselves to be the testing ground for new approaches to engage patients in their care.

Answer choices were assigned values of Never = 0, Sometimes = 33.33, Often = 66.66, and Always = 100. Composite scale scores were obtained by calculating the mean of the 5 items; scale scores could range from 0-100.

(2) **Advanced information systems.** Respondents were asked whether the practice's health information system (including the EHR) allows the following (Answer choices: Yes or No).

- Practice EHR connects with EHR at the main hospital.
- Patients to have electronic access to their medical records.
- Patients to electronically comment on and/or input information to their medical records.
- Secure messaging between physicians and patients.
- Physicians to know whether their patients have filled prescriptions.
- Advanced analytic systems such as predicting future utilization, data mining, etc.

To calculate composite scale scores, we assigned values of No = 0, Yes = 100. Composite scale scores were obtained by calculating the mean of the 6 items (possible range: 0-100).

(3) **Exposure to value-based payment models.** Respondents were asked whether the practice had ever participated in the following payment models (Answer choices: Yes, currently; Yes, previously but not now; No, never).

- Capitated contracts with commercial health plans.
- Medicare ACO contracts
- Commercial ACO contracts
- Medicaid ACO contracts

To calculate composite scale scores, we assigned values of No = 0; Yes, previously but not now = 50; Yes, currently = 100. Composite scale scores were obtained by calculating the mean of the 4 items (possible range: 0-100).

© 2025 Brewster AL et al. *JAMA Network Open.*

**eTable 1.** Marginal Mean Count of Social Risks Screened From Main Model

| | Marginal mean count of social risks screened | Delta-method Std. Err. | [95% Conf. | Interval] |
|---|---|---|---|---|
| Year _at | | | | |
| 2017 | 1.68 | 0.05 | 1.59 | 1.78 |
| 2022 | 2.39 | 0.09 | 2.21 | 2.58 |
| Share of practice revenue from Medicaid _at | | | | |
| <10% | 1.94 | 0.07 | 1.79 | 2.08 |
| 10-19% | 1.81 | 0.13 | 1.55 | 2.06 |
| 20-49% | 2.04 | 0.09 | 1.87 | 2.22 |
| 50-79% | 2.25 | 0.16 | 1.94 | 2.56 |
| >80% | 2.21 | 0.25 | 1.73 | 2.70 |
| Innovation Culture Score _at | | | | |
| 0 | 1.05 | 0.07 | 0.90 | 1.19 |
| 10 | 1.18 | 0.07 | 1.04 | 1.32 |
| 20 | 1.34 | 0.07 | 1.21 | 1.47 |
| 30 | 1.51 | 0.06 | 1.39 | 1.63 |
| 40 | 1.71 | 0.06 | 1.60 | 1.82 |
| 50 | 1.93 | 0.05 | 1.83 | 2.03 |
| 60 | 2.18 | 0.06 | 2.07 | 2.29 |
| 70 | 2.47 | 0.07 | 2.33 | 2.61 |
| 80 | 2.79 | 0.10 | 2.59 | 2.98 |
| 90 | 3.15 | 0.14 | 2.87 | 3.43 |
| 100 | 3.56 | 0.20 | 3.18 | 3.94 |
| Advanced Information Systems Score _at | | | | |
| 0 | 1.64 | 0.11 | 1.43 | 1.86 |
| 10 | 1.69 | 0.10 | 1.50 | 1.89 |
| 20 | 1.75 | 0.08 | 1.58 | 1.91 |
| 30 | 1.80 | 0.07 | 1.66 | 1.94 |
| 40 | 1.86 | 0.06 | 1.74 | 1.97 |
| 50 | 1.92 | 0.05 | 1.82 | 2.01 |
| 60 | 1.97 | 0.05 | 1.88 | 2.07 |
| 70 | 2.04 | 0.06 | 1.92 | 2.15 |
| 80 | 2.10 | 0.07 | 1.96 | 2.24 |
| 90 | 2.16 | 0.09 | 1.98 | 2.35 |
| 100 | 2.23 | 0.12 | 2.01 | 2.46 |
| Payment Reform Exposure Score _at | | | | |
| 0 | 1.79 | 0.08 | 1.63 | 1.96 |
| 10 | 1.83 | 0.07 | 1.68 | 1.97 |
| 20 | 1.86 | 0.06 | 1.73 | 1.99 |
| 30 | 1.90 | 0.06 | 1.79 | 2.01 |
| 40 | 1.93 | 0.05 | 1.83 | 2.04 |
| 50 | 1.97 | 0.05 | 1.88 | 2.07 |
| 60 | 2.01 | 0.05 | 1.91 | 2.11 |
| 70 | 2.05 | 0.06 | 1.94 | 2.16 |
| 80 | 2.09 | 0.07 | 1.96 | 2.22 |
| 90 | 2.13 | 0.08 | 1.97 | 2.29 |
| 100 | 2.17 | 0.10 | 1.99 | 2.36 |

© 2025 Brewster AL et al. *JAMA Network Open.*

**eTable 2.** Sensitivity Analysis 1
Change in number of social risks screened within practice associated with changes in time-varying practice characteristics (fixed effects models using cohort of 620 practices surveyed at both time points)

| | **IRR** | **[95% Conf.** | **Interval]** | **p-value** |
|---|---|---|---|---|
| Year | | | | |
| 2017 | (reference) | | | |
| 2022 | 1.478*** | 1.353 | 1.614 | 0.000 |
| Share of practice revenue from Medicaid | | | | |
| <10% | (reference) | | | |
| 10-19% | 0.787** | 0.669 | 0.926 | 0.004 |
| 20-49% | 0.873 | 0.731 | 1.042 | 0.131 |
| 50-79% | 0.966 | 0.736 | 1.268 | 0.804 |
| >80% | 0.718 | 0.433 | 1.191 | 0.200 |
| Innovation culture score | 1.009*** | 1.006 | 1.012 | 0.000 |
| Advanced information systems score | 1.005** | 1.002 | 1.008 | 0.001 |
| Payment reform score | 0.999 | 0.998 | 1.001 | 0.516 |

IRR (incidence rate ratio) values are exponentiated coefficients which reflect the count of social risk screenings per practice associated with the independent variables. Reported screenings can range from 0 to 5. A total of 714 practices responded to the survey in both 2017 and 2022, however, 94 practices had reported zero social risk screenings in both years and therefore were dropped from the model.

$^{*}$ $p < 0.05$, $^{**}$ $p < 0.01$, $^{***}$ $p < 0.001$

© 2025 Brewster AL et al. *JAMA Network Open.*

**eTable 3.** Sensitivity Analysis 2

Poisson model of count of social risks screened, plus interaction terms for year x practice characteristics

| | Model 1 | | Model 2 | | Model 3 | | Model 4 | | Model 5 | |
|---|---|---|---|---|---|---|---|---|---|---|
| **Interaction of:** | **Year X ownership** | | **Year X revenue from Medicaid** | | **Year X exposure to payment reform** | | **Year X culture of innovation** | | **Year X advanced information systems** | |
| | IRR | p-value | IRR | p-value | IRR | p-value | IRR | p-value | IRR | p-value |
| 2017.year | 1 | (.) | 1 | (.) | 1 | (.) | 1 | (.) | 1 | (.) |
| 2022.year | 1.214$^{*}$ | (0.050) | 1.365$^{***}$ | (0.000) | 1.294$^{**}$ | (0.005) | 1.712$^{***}$ | (0.000) | 1.469$^{**}$ | (0.004) |
| 1.ownership | 1 | (.) | 1 | (.) | 1 | (.) | 1 | (.) | 1 | (.) |
| 2.ownership | 0.999 | (0.990) | 1.043 | (0.636) | 1.048 | (0.605) | 1.042 | (0.647) | 1.043 | (0.641) |
| 3.ownership | 1.023 | (0.835) | 1.165 | (0.086) | 1.161 | (0.092) | 1.153 | (0.107) | 1.161 | (0.093) |
| 4.ownership | 0.973 | (0.759) | 1.046 | (0.525) | 1.046 | (0.522) | 1.046 | (0.529) | 1.048 | (0.511) |
| 5.ownership | 1.442$^{***}$ | (0.000) | 1.542$^{***}$ | (0.000) | 1.562$^{***}$ | (0.000) | 1.535$^{***}$ | (0.000) | 1.549$^{***}$ | (0.000) |
| 6.ownership | 1.002 | (0.994) | 1.159 | (0.342) | 1.175 | (0.304) | 1.146 | (0.382) | 1.159 | (0.346) |
| 2017.year#1.ownership | 1 | (.) | | | | | | | | |
| 2017.year#2.ownership | 1 | (.) | | | | | | | | |
| 2017.year#3.ownership | 1 | (.) | | | | | | | | |
| 2017.year#4.ownership | 1 | (.) | | | | | | | | |
| 2017.year#5.ownership | 1 | (.) | | | | | | | | |
| 2017.year#6.ownership | 1 | (.) | | | | | | | | |
| 2022.year#1.ownership | 1 | (.) | | | | | | | | |
| 2022.year#2.ownership | 1.121 | (0.557) | | | | | | | | |
| 2022.year#3.ownership | 1.348 | (0.082) | | | | | | | | |
| 2022.year#4.ownership | 1.200 | (0.192) | | | | | | | | |
| 2022.year#5.ownership | 1.203 | (0.162) | | | | | | | | |
| 2022.year#6.ownership | 1.550 | (0.112) | | | | | | | | |
| 1.medicaid_cat | 1 | (.) | 1 | (.) | 1 | (.) | 1 | (.) | 1 | (.) |
| 2.medicaid_cat | 0.931 | (0.372) | 0.897 | (0.192) | 0.926 | (0.331) | 0.928 | (0.352) | 0.932 | (0.378) |
| 3.medicaid_cat | 1.054 | (0.378) | 1.001 | (0.985) | 1.052 | (0.385) | 1.056 | (0.355) | 1.055 | (0.368) |
| 4.medicaid_cat | 1.159 | (0.071) | 1.126 | (0.338) | 1.150 | (0.083) | 1.167 | (0.059) | 1.161 | (0.068) |
| 5.medicaid_cat | 1.132 | (0.319) | 1.393$^{*}$ | (0.018) | 1.132 | (0.305) | 1.151 | (0.238) | 1.140 | (0.277) |
| 1.practice_size | 1 | (.) | 1 | (.) | 1 | (.) | 1 | (.) | 1 | (.) |
| 2.practice_size | 0.936 | (0.273) | 0.940 | (0.301) | 0.938 | (0.281) | 0.940 | (0.299) | 0.935 | (0.259) |
| 3.practice_size | 1.016 | (0.850) | 1.020 | (0.805) | 1.019 | (0.816) | 1.021 | (0.801) | 1.020 | (0.811) |
| 4.practice_size | 0.960 | (0.645) | 0.961 | (0.651) | 0.951 | (0.579) | 0.963 | (0.670) | 0.957 | (0.623) |
| 5.practice_size | 1.038 | (0.608) | 1.038 | (0.615) | 1.037 | (0.622) | 1.036 | (0.631) | 1.037 | (0.620) |
| 1.cen_region | 1 | (.) | 1 | (.) | 1 | (.) | 1 | (.) | 1 | (.) |

© 2025 Brewster AL et al. *JAMA Network Open.*

| | | | | | | | | | | |
|---|---|---|---|---|---|---|---|---|---|---|
| 2.cen_region | 1.003 | (0.967) | 0.995 | (0.947) | 1.000 | (0.996) | 0.997 | (0.963) | 0.996 | (0.957) |
| 3.cen_region | 0.871 | (0.051) | 0.870$^{*}$ | (0.049) | 0.868$^{*}$ | (0.044) | 0.870$^{*}$ | (0.049) | 0.868$^{*}$ | (0.045) |
| 4.cen_region | 0.935 | (0.337) | 0.936 | (0.340) | 0.933 | (0.314) | 0.930 | (0.300) | 0.933 | (0.321) |
| inn_cult | 1.012$^{***}$ | (0.000) | 1.012$^{***}$ | (0.000) | 1.012$^{***}$ | (0.000) | 1.014$^{***}$ | (0.000) | 1.012$^{***}$ | (0.000) |
| adv_is | 1.003$^{**}$ | (0.006) | 1.003$^{**}$ | (0.005) | 1.003$^{**}$ | (0.006) | 1.003$^{**}$ | (0.005) | 1.003$^{*}$ | (0.021) |
| pay_reform | 1.002$^{**}$ | (0.009) | 1.002$^{*}$ | (0.013) | 1.001 | (0.339) | 1.002$^{*}$ | (0.013) | 1.002$^{*}$ | (0.011) |
| 2017.year#1.medicaid_cat | | | 1 | (.) | | | | | | |
| 2017.year#2.medicaid_cat | | | 1 | (.) | | | | | | |
| 2017.year#3.medicaid_cat | | | 1 | (.) | | | | | | |
| 2017.year#4.medicaid_cat | | | 1 | (.) | | | | | | |
| 2017.year#5.medicaid_cat | | | 1 | (.) | | | | | | |
| 2022.year#1.medicaid_cat | | | 1 | (.) | | | | | | |
| 2022.year#2.medicaid_cat | | | 1.081 | (0.621) | | | | | | |
| 2022.year#3.medicaid_cat | | | 1.119 | (0.338) | | | | | | |
| 2022.year#4.medicaid_cat | | | 1.079 | (0.598) | | | | | | |
| 2022.year#5.medicaid_cat | | | 0.763 | (0.175) | | | | | | |
| 2017.year#c.pay_reform | | | | | 1 | (.) | | | | |
| 2022.year#c.pay_reform | | | | | 1.002 | (0.181) | | | | |
| 2017.year#c.inn_cult | | | | | | | 1 | (.) | | |
| 2022.year#c.inn_cult | | | | | | | 0.997 | (0.122) | | |
| 2017.year#c.adv_is | | | | | | | | | 1 | (.) |
| 2022.year#c.adv_is | | | | | | | | | 0.999 | (0.803) |
| *N* | 3442 | | 3442 | | 3442 | | 3442 | | 3442 | |

Exponentiated coefficients; *p*-values in parentheses
$^{*}$ $p < 0.05$, $^{**}$ $p < 0.01$, $^{***}$ $p < 0.001$

IRR (incidence rate ratio) values are exponentiated coefficients which reflect the count of social risk screenings per practice associated with the independent variables. Reported screenings can range from 0 to 5. 2,728 unique practices ever responded to the survey, 714 of which responded in both 2017 and 2022. Standard errors are clustered by practice in the models to account for practices that responded in both years.

© 2025 Brewster AL et al. *JAMA Network Open.*

**eTable 4.** Sensitivity Analysis 3

Factors associated with probability of screening for each of the 5 social risks (weighted logistic model)

| | | Model 1 | | Model 2 | | Model 3 | | Model 4 | | Model 5 | |
|---|---|---|---|---|---|---|---|---|---|---|---|
| | | **Food** | | **Housing** | | **Utilities** | | **IPV** | | **Transportation** | |
| | | O.R. | p-value | O.R. | p-value | O.R. | p-value | O.R. | p-value | O.R. | p-value |
| Year | | | | | | | | | | | |
| | 2017 | 1 | (.) | 1 | (.) | 1 | (.) | 1 | (.) | 1 | (.) |
| | 2022 | 2.441*** | (0.000) | 2.491*** | (0.000) | 2.080*** | (0.000) | 1.324* | (0.028) | 2.072*** | (0.000) |
| Share of practice revenue from Medicaid | | | | | | | | | | | |
| | <10% | 1 | (.) | 1 | (.) | 1 | (.) | 1 | (.) | 1 | (.) |
| | 10-19% | 0.746 | (0.111) | 0.859 | (0.416) | 0.821 | (0.318) | 0.889 | (0.495) | 0.939 | (0.725) |
| | 20-49% | 1.138 | (0.451) | 1.002 | (0.990) | 0.854 | (0.363) | 1.274 | (0.126) | 1.239 | (0.163) |
| | 50-79% | 1.382 | (0.182) | 1.512 | (0.081) | 1.049 | (0.858) | 1.188 | (0.465) | 1.870** | (0.008) |
| | >80% | 2.157* | (0.047) | 2.059 | (0.061) | 1.680 | (0.177) | 1.263 | (0.533) | 1.144 | (0.714) |
| Practice owned by: | | | | | | | | | | | |
| | Independently owned | 1 | (.) | 1 | (.) | 1 | (.) | 1 | (.) | 1 | (.) |
| | Larger physician group | 0.969 | (0.889) | 0.938 | (0.777) | 1.114 | (0.644) | 1.121 | (0.532) | 1.065 | (0.767) |
| | Hospital | 0.947 | (0.814) | 0.935 | (0.774) | 1.177 | (0.520) | 2.391*** | (0.000) | 1.229 | (0.335) |
| | Healthcare system | 0.917 | (0.620) | 1.102 | (0.572) | 1.053 | (0.776) | 1.291 | (0.106) | 0.957 | (0.790) |
| | FQHC | 2.222*** | (0.000) | 3.108*** | (0.000) | 2.595*** | (0.000) | 2.151*** | (0.000) | 2.189*** | (0.000) |
| | Other/Missing | 1.166 | (0.640) | 1.338 | (0.423) | 1.227 | (0.588) | 1.232 | (0.549) | 1.362 | (0.394) |
| Practice size (number of physicians) | | | | | | | | | | | |
| | 0-3 | 1 | (.) | 1 | (.) | 1 | (.) | 1 | (.) | 1 | (.) |
| | 4-7 | 0.877 | (0.406) | 0.860 | (0.334) | 0.859 | (0.344) | 0.830 | (0.213) | 0.909 | (0.528) |
| | 8-12 | 1.068 | (0.762) | 1.226 | (0.361) | 1.177 | (0.473) | 1.033 | (0.874) | 0.818 | (0.343) |
| | 13-19 | 0.857 | (0.538) | 0.839 | (0.474) | 0.764 | (0.312) | 0.874 | (0.602) | 1.206 | (0.449) |
| | 20+ | 1.109 | (0.635) | 1.042 | (0.852) | 0.815 | (0.388) | 1.223 | (0.272) | 1.203 | (0.368) |
| Region | | | | | | | | | | | |
| | Northeast | 1 | (.) | 1 | (.) | 1 | (.) | 1 | (.) | 1 | (.) |
| | Midwest | 0.945 | (0.760) | 0.941 | (0.748) | 0.927 | (0.703) | 1.153 | (0.426) | 0.978 | (0.904) |
| | South | 0.685* | (0.041) | 0.798 | (0.237) | 0.593** | (0.006) | 0.863 | (0.390) | 0.842 | (0.331) |
| | West | 0.833 | (0.333) | 0.896 | (0.558) | 0.741 | (0.125) | 0.998 | (0.991) | 0.811 | (0.252) |
| | | | | | | | | | | | |
| Innovation culture score | | 1.024*** | (0.000) | 1.026*** | (0.000) | 1.027*** | (0.000) | 1.022*** | (0.000) | 1.025*** | (0.000) |

© 2025 Brewster AL et al. *JAMA Network Open.*

| | | | | | | | | | | | |
|---|---|---|---|---|---|---|---|---|---|---|---|
| Advanced information systems score | | 1.009** | (0.001) | 1.005* | (0.049) | 1.005 | (0.095) | 1.007** | (0.005) | 1.003 | (0.218) |
| Payment reform score | | 1.006** | (0.004) | 1.005* | (0.016) | 1.004 | (0.061) | 1.003 | (0.145) | 1.003 | (0.104) |
| *N* | | 3442 | | 3442 | | 3442 | | 3442 | | 3442 | |

O.R. (Odds Ratios) are exponentiated coefficients from each model; *p*-values in parentheses. 2,728 unique practices ever responded to the survey, 714 of which responded in both 2017 and 2022. Standard errors are clustered by practice in the models to account for practices that responded in both years.

* $p < 0.05$, ** $p < 0.01$, *** $p < 0.001$

© 2025 Brewster AL et al. *JAMA Network Open.*
