# Supplementary material for: Trends in Screening for Social Risk in US Physician Practices
Source: JAMA Netw Open. 2025 Jan 3;8(1):e2453117. doi: 10.1001/jamanetworkopen.2024.53117 (PMC11699528; doi:10.1001/jamanetworkopen.2024.53117)
Supplement: Supplement 2. — Data Sharing Statement [file jamanetwopen-e2453117-s002.pdf]

Data Sharing Statement

Brewster. Trends in Screening for Social Risk in US Physician Practices. *JAMA Netw Open*. Published January 03, 2025. doi:10.1001/jamanetworkopen.2024.53117

Data
Data available: No
